# Supplementary material for: A paired sequence language model for protein-protein interaction modeling
Source: Nat Commun. 2026 Mar 10;17:3733. doi: 10.1038/s41467-026-70457-5 (PMC13103355; doi:10.1038/s41467-026-70457-5)
Supplement: Supplementary file 2 — Description of Additional Supplementary Files [file 41467_2026_70457_MOESM2_ESM.pdf]

Description of Additional Supplementary Files

Supplementary Data 1. Precision of inter-protein contact prediction by PPLM-Contact, DeepInter, CDPred, DeepHomo2.0, and GLINTER on the Homodimer300 and CASP_Homodimer43 test sets.

Supplementary Data 2. Precision of inter-protein contact prediction by PPLM-Contact, DeepInter, CDPred, DeepHomo2.0, and GLINTER on the Heterodimer99 and CASP_Heterodimer20 test sets.

Supplementary Data 3. Precision of inter-protein contact prediction of PPLM-Contact2, AlphaFold2.3, AlphaFold3, and DMFold on the Homodimer300, Heterodimer99 and CASP_Homodimer43, and CASP_Heterodimer20 test sets.

Supplementary Data 4. Interface contact prediction accuracy, recall, and F1-score of AlphaFold2.3, AlphaFold3, and DMFold, as well as those of PPLM-Contact2 evaluated under different contact probability thresholds.

Supplementary Data 5. Precision of interface residue identification by PPLM-Contact, DeepInter, CDPred, DeepHomo2.0, GLINTER, PPLM-Contact2, AlphaFold2.3, AlphaFold3, and DMFold on the 343 homodimer test proteins.

Supplementary Data 6. Precision of interface residue identification by PPLM-Contact, DeepInter, CDPred, DeepHomo2.0, GLINTER, PPLM-Contact2, AlphaFold2.3, AlphaFold3, and DMFold on the 119 heterodimer test proteins.

Supplementary Data 7. List of PDB IDs used for five-fold cross-validation of PPLM-Affinity.
