# Supplementary material for: A paired sequence language model for protein-protein interaction modeling
Source: Nat Commun. 2026 Mar 10;17:3733. doi: 10.1038/s41467-026-70457-5 (PMC13103355; doi:10.1038/s41467-026-70457-5)
Supplement: Supplementary file 4 — Reporting Summary [file 41467_2026_70457_MOESM4_ESM.pdf]

nature portfolio

Corresponding author(s): Yang Zhang

Last updated by author(s): Feb 13, 2026

Reporting Summary

Nature Portfolio wishes to improve the reproducibility of the work that we publish. This form provides structure for consistency and transparency in reporting. For further information on Nature Portfolio policies, see our Editorial Policies and the Editorial Policy Checklist.

Statistics

For all statistical analyses, confirm that the following items are present in the figure legend, table legend, main text, or Methods section.

n/a	Confirmed	
☐	☒	The exact sample size (*n*) for each experimental group/condition, given as a discrete number and unit of measurement
☐	☒	A statement on whether measurements were taken from distinct samples or whether the same sample was measured repeatedly
☐	☒	The statistical test(s) used AND whether they are one- or two-sided<br>*Only common tests should be described solely by name; describe more complex techniques in the Methods section.*
☒	☐	A description of all covariates tested
☐	☒	A description of any assumptions or corrections, such as tests of normality and adjustment for multiple comparisons
☐	☒	A full description of the statistical parameters including central tendency (e.g. means) or other basic estimates (e.g. regression coefficient) AND variation (e.g. standard deviation) or associated estimates of uncertainty (e.g. confidence intervals)
☐	☒	For null hypothesis testing, the test statistic (e.g. *F*, *t*, *r*) with confidence intervals, effect sizes, degrees of freedom and *P* value noted<br>*Give P values as exact values whenever suitable.*
☒	☐	For Bayesian analysis, information on the choice of priors and Markov chain Monte Carlo settings
☒	☐	For hierarchical and complex designs, identification of the appropriate level for tests and full reporting of outcomes
☒	☐	Estimates of effect sizes (e.g. Cohen's *d*, Pearson's *r*), indicating how they were calculated

Our web collection on statistics for biologists contains articles on many of the points above.

Software and code

Policy information about availability of computer code

Data collection	Protein complex structures were retrieved from the Protein Data Bank (PDB; accessed January 2024). High-confidence protein–protein interaction (PPI) pairs were obtained from the STRING database (version 11.5). Sequence clustering was performed using MMseqs2 (commit ID: aa175d63658d9aa2e908325a6fd40e9dbb260c9a). The PPI benchmark dataset was sourced from D-SCRIPT (version 1.0; https://github.com/samsledje/D-SCRIPT/), and the binding affinity dataset was obtained from PPB-Affinity (version 1.2; https://github.com/ChenPy00/PPB-Affinity). The UniRef30_2021_03 database (version March 2021; https://wwwuser.gwdg.de/~compbiol/uniclust/) was used for MSA search.
Data analysis	HHblits (3.3.0), CCMPred (1.0), LoadHMM (1.0), and ESM-MSA (esm_msa1b_t12_100M_UR50S) were used to generate features for inter-protein contact prediction. ESM2 (esm2_t36_3B_UR50D) was used to train ESM2-Affinity. AlphaFold2 (2.2.4), AlphaFold3, and DMFold (1.2) were used to generate monomer or complex protein structures.

For manuscripts utilizing custom algorithms or software that are central to the research but not yet described in published literature, software must be made available to editors and reviewers. We strongly encourage code deposition in a community repository (e.g. GitHub). See the Nature Portfolio guidelines for submitting code & software for further information.

Data

Policy information about availability of data

All manuscripts must include a data availability statement. This statement should provide the following information, where applicable:

- Accession codes, unique identifiers, or web links for publicly available datasets
- A description of any restrictions on data availability
- For clinical datasets or third party data, please ensure that the statement adheres to our policy

All datasets used in this study are publicly available. The benchmark datasets and processed features are deposited at both our project website (https://zhanggroup.org/PPLM/benchmark.html) and GitHub repository (https://github.com/junliu621/PPLM/tree/main/data/). Protein sequences and structures used to train PPLM were collected from the Protein Data Bank (PDB; https://www.rcsb.org/) and STRING database (https://string-db.org/). The interaction dataset for PPLM-PPI was obtained from D-SCRIPT (https://github.com/samsledje/D-SCRIPT/), and the binding affinity dataset for PPLM-Affinity from PPB-Affinity (https://github.com/ChenPy00/PPB-Affinity). The Uniref30_2021_03 database used for MSA generation is available at https://wwwuser.gwdg.de/~compbiol/uniclust/. There are no restrictions on data availability.

Research involving human participants, their data, or biological material

Policy information about studies with human participants or human data. See also policy information about sex, gender (identity/presentation), and sexual orientation and race, ethnicity and racism.

Reporting on sex and gender	NA
Reporting on race, ethnicity, or other socially relevant groupings	NA
Population characteristics	NA
Recruitment	NA
Ethics oversight	NA

Note that full information on the approval of the study protocol must also be provided in the manuscript.

Field-specific reporting

Please select the one below that is the best fit for your research. If you are not sure, read the appropriate sections before making your selection.

[x] Life sciences [ ] Behavioural & social sciences [ ] Ecological, evolutionary & environmental sciences

For a reference copy of the document with all sections, see nature.com/documents/nr-reporting-summary-flat.pdf

Life sciences study design

All studies must disclose on these points even when the disclosure is negative.

Sample size	To train and evaluate the protein–protein language model, we collected over 3.3 million protein sequence pairs from the PDB and STRING databases. These pairs were clustered into 677,372 non-redundant groups based on sequence similarity to reduce redundancy. From these clusters, 5,000 protein pairs were randomly selected as the validation set, while the remaining pairs were used for training. For protein–protein interaction (PPI) prediction, the training set comprises 43,138 positive and 430,947 negative samples from Homo sapiens. The test set includes 22,000 positive and 216,183 negative samples drawn from five different species, enabling cross-species evaluation. For binding affinity prediction, the dataset contains 12,062 interaction samples derived from 3,032 distinct PDB entries, encompassing a wide range of complex types, including antibody–antigen and TCR–pMHC interactions. A five-fold cross-validation strategy was employed, with samples split at the complex level to prevent data leakage across folds. For inter-protein contact prediction, the training set consists of 3,504 non-redundant homodimers and 1,881 non-redundant heterodimers, while the validation set includes 296 homodimers and 96 heterodimers. The test set contains 300 homodimers and 99 heterodimers. To further assess generalizability, an independent test set comprising 43 homodimers and 20 heterodimers was curated from CASP13 to CASP16.
Data exclusions	To ensure data quality, we removed a total of 4,319 samples from the D-SCRIPT PPI datasets, including 4,163 duplicate, 57 erroneous, and 31 invalid samples. These samples were excluded across six species: H. sapiens (432 samples), M. musculus (1), D. melanogaster (7), C. elegans (4), S. cerevisiae (37), and E. coli (3,768).
Replication	Al results could be reproduced by our server and standalone package with the full version databases or based on the information provided in manuscript. All experiments are done independently without any technical replication.
Randomization	For training and evaluation of the language model, the test set was randomly split from the non-redundant dataset to ensure unbiased performance assessment. For the downstream tasks of protein-protein interaction prediction, binding affinity estimation, and interface contact prediction, benchmark datasets were adopted from previously published studies or standard evaluation benchmarks. In these datasets, the allocation of samples into training, validation, and test sets was predefined and widely accepted in the field.
Blinding	Blinding was not performed, as all samples were derived from pre-existing public datasets with predefined labels. The data were preprocessed

to remove redundancy and ensure no information leakage between training and testing sets. The analysis was conducted using automated computational methods, minimizing the risk of subjective bias.

Reporting for specific materials, systems and methods

We require information from authors about some types of materials, experimental systems and methods used in many studies. Here, indicate whether each material, system or method listed is relevant to your study. If you are not sure if a list item applies to your research, read the appropriate section before selecting a response.

Materials & experimental systems

n/a	Involved in the study
x	Antibodies
x	Eukaryotic cell lines
x	Palaeontology and archaeology
x	Animals and other organisms
x	Clinical data
x	Dual use research of concern
x	Plants

Methods

n/a	Involved in the study
x	ChIP-seq
x	Flow cytometry
x	MRI-based neuroimaging

Plants

Seed stocks	NA
Novel plant genotypes	NA
Authentication	NA
